# Identification of the SbDUF966 Gene Family in Sorghum and Investigation of It Role in Response to Abiotic Stresses

**DOI:** 10.3390/genes16020206

**Published:** 2025-02-08

**Authors:** Yu Luo, Minli Wang, Wenda Jiao, Kun Huang, Jiaqi Li, Haiyun Chen, Ruidong Zhang, Xiong Cao

**Affiliations:** 1 College of Agronomy, Shanxi Agricultural University, Jinzhong 030801, China; 20233219@stu.sxau.edu.cn (Y.L.); wangminli@sxau.edu.cn (M.W.); 202430160@stu.sxau.edu.cn (W.J.); 20233190@stu.sxau.edu.cn (K.H.); 20233114@stu.sxau.edu.cn (J.L.); z20223074@stu.sxau.edu.cn (H.C.); 2 Institute of Industrial Crops, Shanxi Agricultural University, Taiyuan 030031, China

**Keywords:** sorghum, DUF966 gene family, abiotic stress, expression analysis

## Abstract

Background: Sorghum (*Sorghum bicolor* L.) is an adversity-tolerant crop, but the function of the DUF966 gene family in its growth, development, and stress tolerance is unclear. Methods: The SbDUF966 gene was identified and analyzed using bioinformatics methods in this study. We also analyzed the expression pattern of SbDUF966 in different tissues and stress conditions using RNA-seq and RT-qPCR. We explored its post-transcriptional regulatory mechanism by combining it with miRNA analysis. Results: A total of six SbDUF966 genes were identified and categorized into two groups (Group I and Group II). Gene expression analysis showed that SbDUF966 exhibited specific expression in different tissues and developmental stages, and the expression response to abiotic stresses such as low temperature, high temperature, salinity, and flooding varied over time. In addition, 12 sorghum miRNAs were predicted as potential regulators of SbDUF966. Conclusions: The SbDUF966 gene family likely regulates sorghum’s growth, development, and stress tolerance.

## 1. Introduction

DUF (Domain of Unknown Function) refers to a group of protein families with recognized structural domains but undefined biological functions [1,2]. Despite their conserved structures, the specific roles of many DUF proteins remain unknown [3]. The Pfam database (Version 35.0) records 19,000 protein families, of which nearly 24% are DUF families. These proteins are found in various organisms, including plants, animals, fungi, and bacteria [4]. In recent years, with the in-depth study of plant DUF family members, the functions of some DUF proteins have been gradually revealed, and it was found that the DUF family plays a wide range of roles in key biological processes, such as growth and development, stress response, and other biological processes in plants [3]. Genes containing the conserved DUF538 structural domain have been reported to be important for trichome development and to influence chlorophyll production [5]. The DUF640 family genes, TH1/BSG1, were strongly expressed in the palea of both young inflorescences and spikelets of rice, and the genes play a key positive regulatory role in the development of rice palea, as well as in the shape and size of the seed grain [6]. The expression of the DUF642 gene may be associated with fluctuations in pectin methyl esterase activity or may influence the degree of methyl esterification of homogalacturonic acid during the development of various cell types [7]. Secondly, the DUF642 gene may respond to signaling pathways associated with cell wall development by regulating pectin methyl esterase activity.

Additionally, the DUF family impacts plant fertility. The Arabidopsis DUF761-1 gene regulates plant morphology and reproductive development, and mutations in the DUF1668 gene cause pollen sterility in rice, contributing to hybrid incompatibility [8,9]. The DUF-246 family, involved in pectin biosynthesis, also affects male fertility in Arabidopsis [10]. In tobacco, overexpression of NtDUF868-E5 enhances growth, photosynthesis, and chlorophyll production [11]. DUF genes play important roles in regulating abiotic stress responses. In *Arabidopsis thaliana*, the AtDUF569 knockout mutant shows enhanced resistance, possibly due to the upregulation of SA-dependent PR genes, which mitigate disease symptoms [12]. In rice, the stress-induced DUF1644 protein (Os-SIDP366) enhances drought and salt tolerance [13]. The DUF1645-containing gene OsSGL, when expressed in rice and Arabidopsis, significantly boosts drought tolerance and alters the expression of stress-related genes [14]. Additionally, OsDUF872.2 enhances Escherichia coli resistance to heat stress. At the same time, overexpression of OsDUF6 triggers changes in growth hormone and receptor-like kinase genes under salt stress, acting as a positive regulator of salt tolerance in rice [15,16].

The DUF966 (Domain of Unknown Function 966) gene family is a part of the large DUF protein group, which is increasingly studied due to its widespread presence in plants. Members of the DUF966 family are involved in regulating plant growth, development, and stress responses. For example, CsDUF966_4c and CsDUF966_7c play key roles in cucumber fruit development [17]. In Arabidopsis, At3g46110 (At Aux RP3) influences leaf development and inflorescence initiation by regulating growth hormone synthesis [18]. In wheat, the TaDUF966 gene is induced by salt stress, with TaDUF966-9B confirmed as important under such conditions through virus-induced gene silencing (VIGS) [19]. In rice, overexpression of OsDSR2, a DUF966-containing gene, increased salt and simulated drought sensitivity while reducing ABA sensitivity [20]. Conversely, OsDSR3 enhances alkali stress tolerance and is linked to chlorophyll, sucrose, and carotenoid metabolism [21].

Sorghum is a widely grown graminaceous plant with remarkable stress tolerance, including salinity, heat, and drought tolerance [22,23]. Due to its unique resistance, it is often arranged to be planted in marginal lands. As a result, sorghum is also frequently subjected to some abiotic stresses during its growth and development. The DUF966 family’s critical function in plant stress tolerance has drawn much attention. However, relatively few studies have been conducted on the DUF966 family of genes in sorghum, limiting our understanding of the potential roles of the gene family in sorghum stress tolerance. In this study, bioinformatics analysis was performed to identify and characterize the DUF966 gene family in sorghum and to investigate its role in stress responses. The findings provide a scientific basis for a deeper understanding of the mechanisms underlying sorghum’s stress tolerance and its potential for genetic improvement.

## 2. Materials and Methods

### 2.1. Identification of Sorghum SbDUF966 Gene Family and Analysis of Chromosomal Localization

To identify the members of the SbDUF966 gene family in sorghum, the genome and annotation files of sorghum BTx623 (ID: 4558) were first obtained from the NCBI database (https://www.ncbi.nlm.nih.gov/ (accessed on 5 July 2024)). The DUF966 protein sequence in Arabidopsis was obtained from Ensembl Plant (https://plants.ensembl.org/index.html (accessed on 5 July 2024)). Then, Blastp comparison was performed using Tbtools (v2.152) software to initially obtain the protein sequences of sorghum DUF966 gene family members using the Arabidopsis DUF966 protein sequence as a reference with the screening threshold set to E-value < 1 × 10^−10^. Secondary screening was performed through the Pfam database (https://www.ebi.ac.uk/interpro/entry/pfam (accessed on 5 July 2024)) to confirm the candidate protein sequence of (PF06136) containing the DUF966 structural domain, and the sorghum DUF966 gene family sequence was extracted using the HMMER tool (https://www.ebi.ac.uk/Tools/hmmer/ (accessed on 5 July 2024)). Combining the intersection of local Blastp and HMMER results, redundant parts of the sequences were removed using the CD-HIT (4.8.1) tool to ensure that unique protein sequences were obtained. Finally, the screened protein sequences were submitted to the SMART database (https://smart.embl.de/ (accessed on 8 July 2024)) for manual identification, and sequences lacking the DUF966 structural domain were eliminated to verify the members of the sorghum DUF966 gene. Finally, the genes were sequentially named SbDUF966-1 to SbDUF966-6 based on the chromosomal location of the gene from top to bottom.

### 2.2. Analysis of SbDUF966 Chromosome Localization and Physicochemical Properties

The positional information of the SbDUF966 gene family members was extracted from the annotation information (GFF3 file) of the sorghum genome and visualized using Tbtools software to show the distribution of SbDUF966 gene family members in the genome. The CDS (coding sequence) of each SbDUF966 gene was analyzed using the ExPasy website (https://web.expasy.org/protparam/ (accessed on 15 July 2024)) to identify its physicochemical properties such as CDS length, protein length, molecular weight, isoelectric point, and hydrophilicity, in order to assess its functional characteristics and physicochemical properties. To further investigate the sequence conservation and variation in the SbDUF966 family members, multiple sequence comparison was performed using MEGA 11.0 software. The comparison results will be visualized by ESPript 3.0 (https://espript.ibcp.fr/ESPript/ESPript/index.php (accessed on 15 July 2024)) to generate intuitive comparison maps showing sequence conservation, variation, and structural domain distribution. This will enhance the comprehension of the evolutionary characteristics and functional variety of the SbDUF966 family.

### 2.3. Analysis of SbDUF966 Gene Structure, Conserved Motifs and Conserved Domains

The “Gene Location Visualize” function in TBtools (v2.152) was used to map the gene structures of the sorghum SbDUF966 gene family members, highlighting their locations and structural features within the genome. The subcellular localization of the SbDUF966 protein was predicted using Cell-PLoc 2.0 (http://www.csbio.sjtu.edu.cn/bioinf/plant-multi/ (accessed on 15 July 2024)) to clarify its potential biological function further. To analyze the conserved structure of the SbDUF966 protein, conserved motifs were identified using an online tool on the MEME website (https://meme-suite.org/meme/tools/meme (accessed on 15 July 2024)), with a parameter setting of 10 motif sets. The conserved structural domains of the SbDUF966 protein were predicted utilizing the NCBI-CDD tool on the NCBI website (https://www.ncbi.nlm.nih.gov/ (accessed on 15 July 2024)) to enhance understanding of the protein’s functional areas, with findings displayed using TBtools (v2.152). Tertiary structure prediction for the SbDUF966 protein was conducted via SWISS-MODEL (https://swissmodel.expasy.org/ (accessed on 20 July 2024)).

### 2.4. Evolutionary Relationship Analysis of SbDUF966

The DUF966 protein sequences of Arabidopsis, *Oryza sativa*, and *Triticum aestivum* were downloaded from the Ensembl Plant database (https://plants.ensembl.org/index.html (accessed on 2 August 2024)). Phylogenetic analysis of the DUF966 protein amino acid sequences of these species was performed using the Neighbor-Joining (NJ) method in the MEGA11.0 software, with the Bootstrap value set to 1000 replicates to ensure the stability of the tree. A phylogenetic tree between sorghum and Arabidopsis, Oryza sativa, and Triticum aestivum was constructed based on this analysis. Finally, the phylogenetic trees were visualized and landscaped using ITOL (https://itol.embl.de/login.cgi (accessed on 3 August 2024)) and Adobe Illustrator 27.1.0 software in order to visualize the evolutionary relationships between different species.

### 2.5. Predictive Analysis of Cis-Elements of the SbDUF966 Promoter

Sequences 2000 bp upstream of the start codon of the sorghum SbDUF966 gene family members were extracted using TBtools software, and cis-elements were analyzed online via the Plant CARE (http://bioinformatics.psb.ugent.be/webtools/plantcare/html/ (accessed on 20 August 2024)) website Analysis. All identified cis-regulatory elements were counted via Excel, and essential promoter elements (e.g., TATA-box and CAAT-box) were eliminated. Subsequently, the analysis results were visualized using the TVBOT (https://www.chiplot.online/tvbot.html (accessed on 21 August 2024)) website to demonstrate the distribution of cis-regulatory elements and potential regulatory features of the sorghum SbDUF966 gene.

### 2.6. Syntenic Analysis of the SbDUF966 Family Genes

First, duplication events of the sorghum SbDUF966 gene were analyzed using TBtools software and visualized by Circos to demonstrate the duplication distribution and structure of SbDUF966 gene family members in the sorghum genome. Subsequently, the Ka/Ks analysis function in TBtools was used to calculate the ratio of synonymous substitutions (Ks) and non-synonymous substitutions (Ka) for each pair of duplicated genes and to assess the selection pressure in gene duplication events. In addition, A. thaliana, O. sativa, *Zea mays*, *Hordeum vulgare*, and *Glycine max* were downloaded from the NCBI website (https://www.ncbi.nlm.nih.gov/ (accessed on 27 August 2024)) for the gene sequences of five plants. The homology between the sorghum SbDUF966 gene and the corresponding genes in the other five plants was analyzed by the MCScanX tool to reveal the evolutionary relationship and gene conservation between them.

### 2.7. Prediction of miRNAs Targeting SbDUF966

To identify potential miRNAs targeting sorghum SbDUF966 transcripts, 12 sorghum mature miRNA sequences were collected from the literature [24]. Then, the CDS sequences of miRNAs and SbDUF966 were uploaded to the psRNATarget online tool (https://www.zhaolab.org/psRNATarget/ (accessed on 27 August 2024)) for analysis. The targeting relationship between miRNAs and SbDUF966 was visualized by origin software to investigate the potential miRNA regulation further.

### 2.8. Analysis of SbDUF966 Gene Expression Pattern in Different Tissues

RNA-Seq data (ERP024508 and PRJNA684417) were downloaded from the ENA database (https://www.ebi.ac.uk/ena/browser/home (accessed on 3 September 2024)) and were used to study the expression pattern of sorghum SbDUF966 gene in different tissues. In the experiment, root, shoot, leaf, and seedling were collected 14 days after germination; embryo, endosperm, and pericarp 20 days after pollination; and pollen at the 9–10 weeks stage. Inflorescences were collected at the following sizes: 1–5 mm (inflorescence-1), 5–10 mm (inflorescence-2), and 1–2 cm (inflorescence-3). All the tissues were immediately frozen in liquid N2. For each tissue, at least 10 plants were pooled in each of three biological replicates. RNA samples were sequenced using an Illumina HiSeq 2500 system (San Diego, CA, USA), producing 250 bp end-pair reads.

### 2.9. Plant Material and Abiotic Stresses

The experimental material used in this study consisted of seeds of sorghum variety “Ji 2055B” kept in our laboratory. The cultivation conditions (growing medium) consisted of a mixture of peat soil, perlite: vermiculite (3:1:1) at a constant temperature of 25 °C, with a photoperiod of 12 h of light/12 h of darkness, with a light intensity of 5000 lux, and relative humidity (RH) maintained between 60% and 70%. Seeds of uniform size and whole grain were selected and sterilized using 75% ethanol for 1 min and then washed thoroughly with distilled water to remove residual ethanol. The cleaned seeds were uniformly sown in nursery pots covered with 2 cm thick vermiculite and irrigated with distilled water until the vermiculite was well absorbed until the sorghum seedlings reached the three-leaf stage. At the three-leaf stage, the seedlings were subjected to flooding, salt, and cold and heat stress treatments. Flooding stress was applied by the two-pot method, salt stress was applied with 150 mM NaCl solution, cold stress was applied at 4 °C, and heat stress was treated at 42 °C. Both cold and heat stresses were carried out in a multifunctional incubator. The uppermost leaves were collected at 0 h, 6 h, 12 h, and 48 h after the start of all the stress treatments, with three biological replicates at each time point. The collected leaves were immediately snap-frozen with liquid nitrogen and stored in an ultra-low temperature refrigerator at −80 °C for subsequent analysis.

### 2.10. qRT-PCR Analysis

RNA from sorghum was extracted using a polysaccharide polyphenol plant RNA extraction kit (Servicebio, China) according to the manufacturer’s instructions. The cDNA was synthesized using 5× SweScript All-in-One SuperMix for qPCR (Servicebio, Wuhan, China), and the synthesized cDNA was stored at −20 °C for backup. The primers were synthesized by Wuhan Sevier Biotechnology Co., Ltd., Wuhan, China (details provided in Table S1 of the Supplementary File). The cDNA was synthesized, and the samples were used for real-time quantitative PCR (RT-qPCR) analysis. 2× Universal Blue SYBR Green qPCR Master Mix (Servicebio, China) was used as the qPCR reaction system. The conditions were set as follows: initial denaturation was performed at 95 °C for 30 s, followed by 40 cycles: denaturation at 95 °C for 15 s, annealing at 60 °C for 30 s, and extension at 60 °C for 30 s; finally, the melting curve was detected by melting curve analysis in the range of 60–95 °C. The total volume of each reaction system was 15.5 mL. The total amount of each reaction system was 15 μL, which included 7.5 μL of the 2× ChamQ Universal SYBR qPCR Master Mix, 2 μL of the diluted cDNA, and 1.5 μL of the forward and reverse primers; the Sb18s rRNA gene was standardized as an internal reference gene. Three biological and technical replicates were performed for all experiments, and the relative gene expression levels were calculated by the 2^−∆∆CT^ method [25].

## 3. Results

### 3.1. Identification and Characterization of the Sorghum SbDUF966 Genes

**Figure 1:**
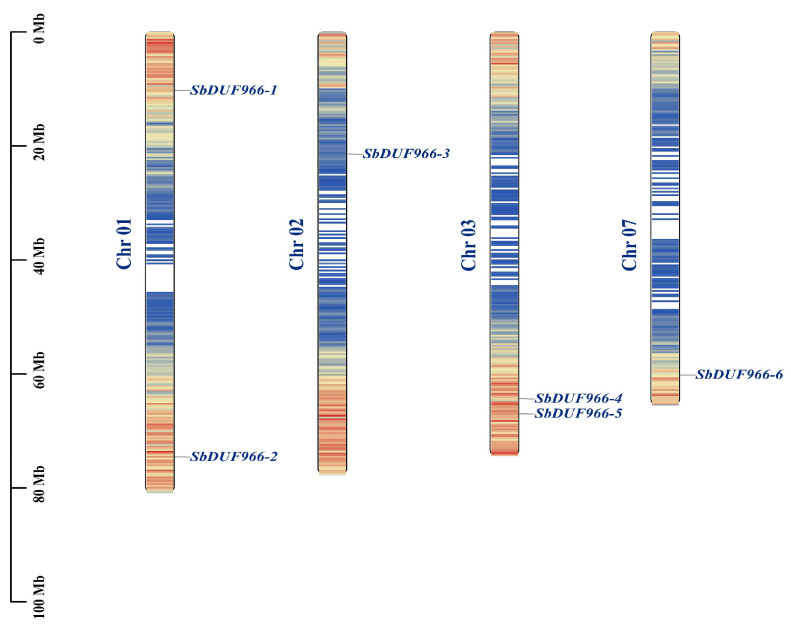
Chromosome distribution of SbDUF966s. The blue font indicates the sorghum chromosome number and the name of the SbDUF966s, and the scale shown in black on the left indicates the length of each chromosome.

To identify the DUF966 genes in the sorghum genome, a combination of Blastp method and HMMER search was used. Six DUF966 genes were successfully identified from the sorghum genome (Table 1). The DUF966 genes were named SbDUF966-1 to SbDUF966-6 based on their locations on the chromosomes. SbDUF966 showed a heterogeneous distribution among the chromosomes, with total distributions of 1, 2, 3, and 7 (Figure 1). Chromosomes 1 and 3 each contained two SbDUF966 genes contained in chromosome 1 and chromosome 3. In contrast, chromosome 2 and chromosome 7 contained only one SbDUF966. Among the six SbDUF966 genes, SbDUF966-6 had the most significant amino acid content with 615 amino acids, whereas the smallest one was SbDUF966-2 with 374 amino acids; according to the molecular weight results, the relative molecular mass of the gene was 40.95 KDa (SbDUF966-2) to 66.24 KDa (SbDUF966-6). The PI values ranged from 7.16 (SbDUF966-2) to 9.44 (SbDUF966-5). The average hydrophobicity of all the SbDUF966 proteins was below 0, signifying that they are hydrophilic proteins. Among the six SbDUF966 proteins, prediction by subcellular localization predictions indicated that all six SbDUF966 proteins were found in the nucleus, with SbDUF966-5 possibly residing in the nucleus or chloroplast.

### 3.2. Phylogenetic Analysis of SbDUF966

**Figure 2:**
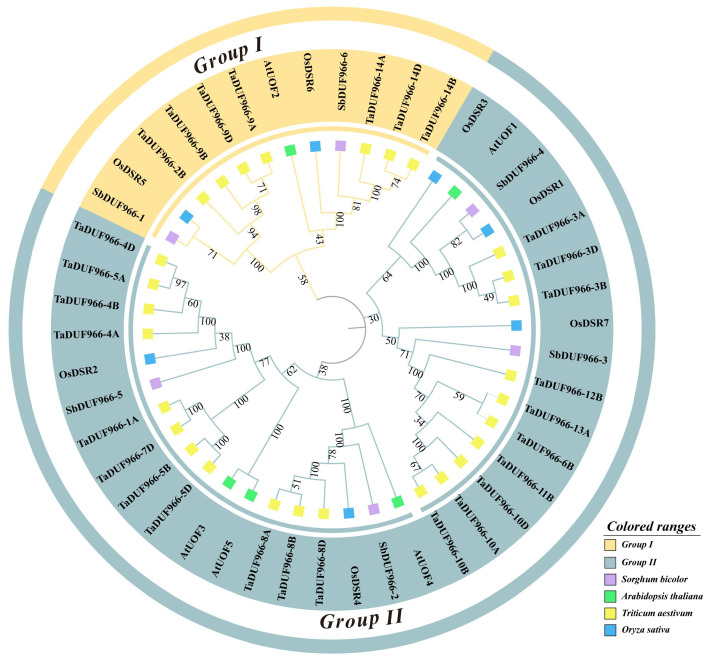
Phylogenetic analysis of DUF966 family members (sorghum, *A. thaliana*, *O. sativa*, and *T. aestivum*). Group I: dark yellow; Group II: gray-blue. Different colored squares represent from sorghum (purple), *A. thaliana* (green), *T. aestivum* (yellow), and *O. sativa* (blue).

To explore the evolutionary and phylogenetic relationships among different species, we used Neighbor-Joining, NJ, of MEGA 11.0 software to construct developmental trees with sorghum, *A. thaliana*, *O. sativa*, and *T. aestivum*, totaling 46 DUF966 proteins. Following the standard classification of Arabidopsis, the 46 DUF966 members were divided into Group I and II (Figure 2). Group I consisted of 12 DUF966 members (1 AtDUF966, 2 SbDUF966, 2 OsSbDUF966, and 7 TaDUF966); Group II contained 34 DUF966 members (4 SbDUF966, 4 AtDUF966, 5 OsSbDUF966, and 21 TaDUF966).

### 3.3. Gene Structure and Motif Composition of SbDUF966

**Figure 3:**
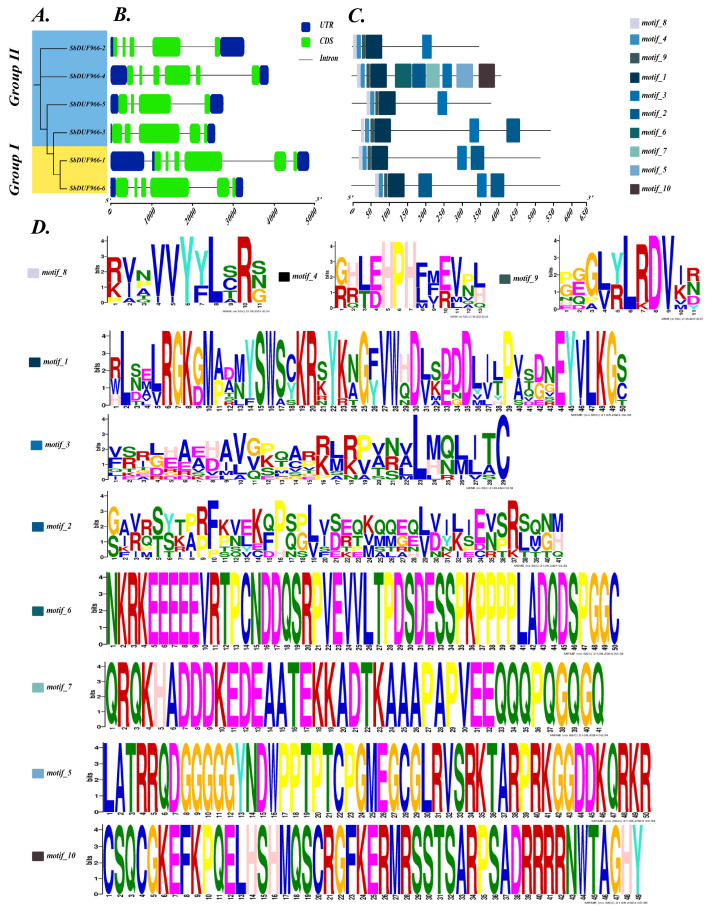
Characterization of SbDUF966. Phylogenetic tree and group (**A**); gene exon/intron structure (**B**); protein motif (**C**); motif sequence (**D**).

The exon/intron structures of SbDUF966 were analyzed to provide insight into the diversification of SbDUF966. The gene structure of SbDUF966 was analyzed by aligning the coding sequence (CDS) of each SbDUF966 gene with the corresponding sorghum genome sequence (Figure 3). The results showed that all the SbDUF966 genes contained introns, with 5–6 introns in two members of Group I and 3–5 introns in four members of Group II. The exon–intron arrangement of the SbDUF966 genes was relatively constant, and the SbDUF966 genes, in particular the homologous SbDUF966, retained relatively high levels of uniformity in their gene structure sequences. It is a specific subset of structural domains that play a role in reflecting and realizing the different biological functions of structural domains. In protein research, the prediction of protein motifs is an important and effective analytical tool that helps to reveal the functions, interactions, and structural features of proteins. By using MEME, a total of 10 motifs were predicted. The results showed that all of these SbDUF966 proteins contain Motif 1, Motif 3, Motif 4, Motif 8, and Motif 9, which are five conserved motifs that might have significant functions in controlling plant growth and development.

### 3.4. Tertiary Structure Prediction and Multiple Sequence Comparison of SbDUF966 Protein

**Figure 4:**
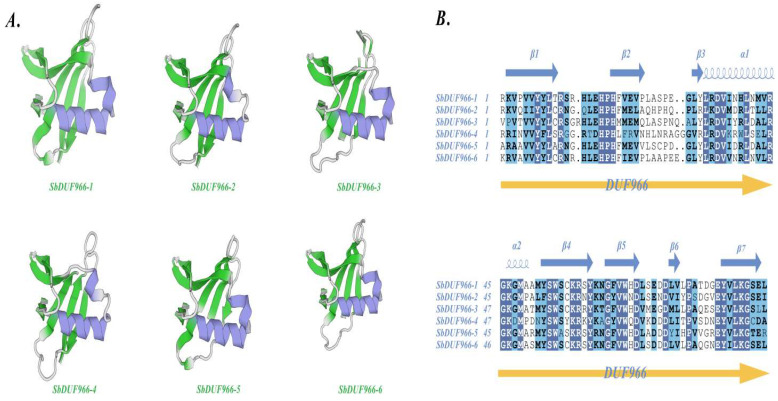
*SbDUF966* protein tertiary structure and multiple sequence comparison. Tertiary structure of SbDUF966 protein (**A**), where green indicates β-folding; blue indicates α-helix. Multiple sequence comparison (**B**).

The 3D model prediction and multiple sequence comparison showed the tertiary structure models and structural domains (Figure 4B) of six members of the SbDUF966 family (SbDUF966-1 to SbDUF966-6), which mainly consist of β-folded lamellae (green) and α-helices (dark blue), and also contain regions of irregularly curled (gray) regions as connecting fragments. As shown in Figure 4A, SbDUF966-1 and SbDUF966-5 exhibited similar 3D structures, whereas SbDUF966-2 and SbDUF966-4 showed similar 3D structures, and SbDUF966-3 and SbDUF966-6 had similar 3D structures. Nonetheless, minor 3D structural differences were observed in the comparatively similar C-termini of the six SbDUF966 proteins.

### 3.5. Analysis of Covariance Between SbDUF966 Gene Duplication Events and Other Species

**Figure 5:**
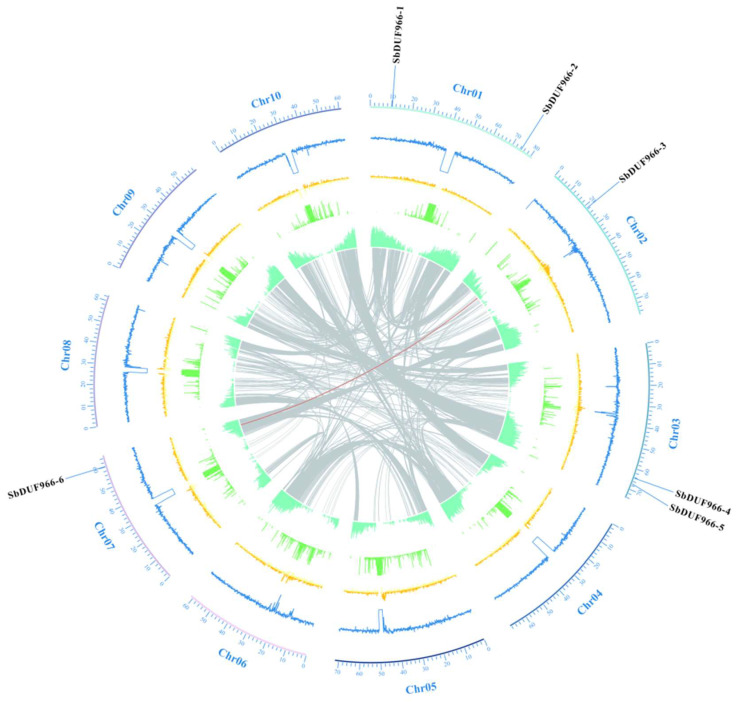
Homozygosity analysis of the sorghum SbDUF966 family. The red curve connecting the SbDUF966 genes indicates duplicate gene pairs in the sorghum SbDUF966 family, and the outer blue font indicates chromosomes.

**Figure 6:**
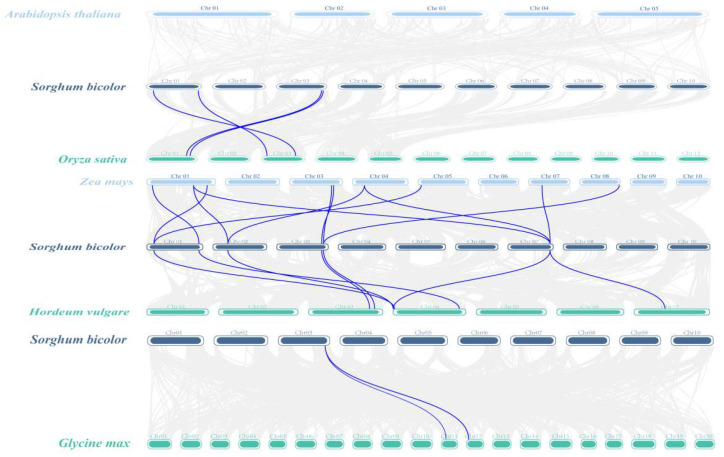
Co-linearity analysis of SbDUF966 genes in sorghum and five different plants. Grey lines in the background indicate blocks of co-linearity in the sorghum and Arabidopsis, rice, maize, barley, and soybean genomes, and the blue line highlights pairs of SbDUF966 genes that are co-linear.

**Figure 7:**
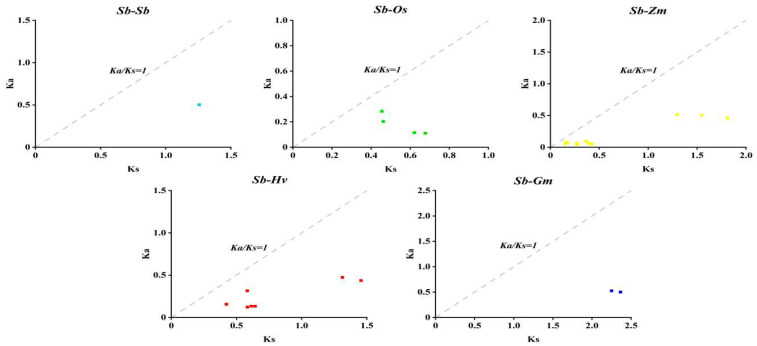
Scatterplot of Ka and Ks of SbDUF966 homologous gene pairs in sorghum and other plants. x-axis indicates Ks and y-axis indicates Ka.

Homologous genes (HGs) are genes with similarity in gene structure and biological function in different species or different genes of the same species due to a common evolutionary origin. Thus, gene duplication plays an indispensable role in determining gene function. One pair of tandemly duplicated paralogs was found in six SbDUF966 genes (Figure 5). To explore the homology of the SbDUF966 genes with those in other plants, we performed covariance analyses to compare the DUF966 homology between monocotyledonous (*O*. *sativa*, *Z*. *mays*, and *H*. *vulgare*) and dicotyledonous (soybean Glycine max and A. thaliana) plants, of DUF966 homology. The findings indicated that 4, 11, 7, and 2 pairs of homologous genes were detected in the comparison of SbDUF966 with OsDUF966, ZmDUF966, HvDUF966, and GmDUF966, respectively (Figure 6). It indicated that the number of DUF966 homologous genes was higher in monocotyledons than in dicotyledons, but no DUF966 homologous genes were found in Arabidopsis. In addition, we assessed the evolutionary selection pressure on the DUF966 gene by calculating the Ka/Ks ratio, which is commonly used to analyze the evolutionary rate of homologous genes or proteins and is an important metric for assessing the evolutionary selection of genes or proteins (Figure 7). Ka/Ks < 1 indicates that the gene is subject to positive selective pressure and may be adapting to new functions or environments; Ka/Ks = 1 indicates a neutral selection, which suggests that the gene is not under significant selection pressure; Ka/Ks > 1 indicates adverse selection, suggesting that the gene is under negative selection, the mutation is not retained, and the gene function is stabilized. The calculated results show that the Ka/Ks ratio of SbDUF966 gene pairs is less than 1, suggesting that these genes have been subjected to positive selection pressure during the evolutionary process.

### 3.6. Analysis of Promoter Cis-Acting Elements of SbDUF966

**Figure 8:**
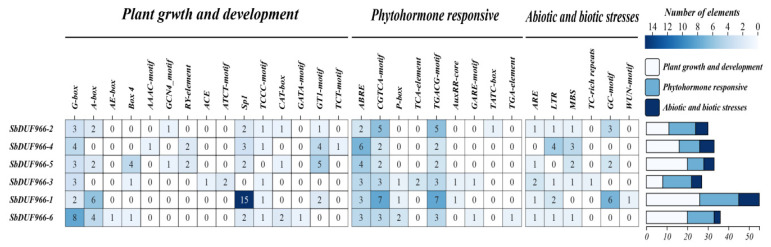
Analysis of cis-acting elements in the SbDUF966 promoter.

Promoter cis-regulatory elements are DNA sequences located in the promoter region of a gene or other regulatory regions that regulate gene transcription by directly binding to transcription factors, thus playing an important role in the regulation of gene expression. To predict the promoter cis-acting elements of the six SbDUF966 genes, we extracted their upstream 2-kb regions and analyzed them using the Plants CARE online tool. A total of 30 cis-acting elements were detected, which can be categorized into three groups: 15 related to plant growth and development, 9 related to hormone response, and 6 related to abiotic and biotic stress response (Figure 8).

Among the plant-growth- and -development-related cis-acting elements, light-responsive elements (Sp1 and G-Box) were the most abundant, appearing 24 and 23 times, respectively, suggesting that light conditions may regulate the expression of the SbDUF966 gene. Among the hormone response elements, several hormone-related elements were detected, including abscisic acid (ABRE), jasmonic acid (TGACG element and CGTCA element), gibberellin (P-box, TATC-box, and GARE element), salicylic acid (TCA element), and growth hormone (TGA element, AuxRR-core element). Among them, abscisic acid (ABRE) and jasmonic acid (TGACG and CGTCA elements) response elements were more significant, suggesting that the SbDUF966 gene may be more responsive to these plant hormones. In addition, we identified multiple abiotic/biotic stress response elements in the SbDUF966 promoter, such as anaerobic-inducible element (ARE, GC motif), low-temperature-responsive element (LTR), drought-inducible element (MBS), defence- and stress-associated sequences (TC-rich repeats), and wound-responsive element (WUN-motif). These findings indicate that the SbDUF966 gene could play a significant role in regulating sorghum’s response to stress conditions.

### 3.7. miRNA-Mediated Regulatory Mechanisms of SbDUF966 Expression

**Figure 9:**
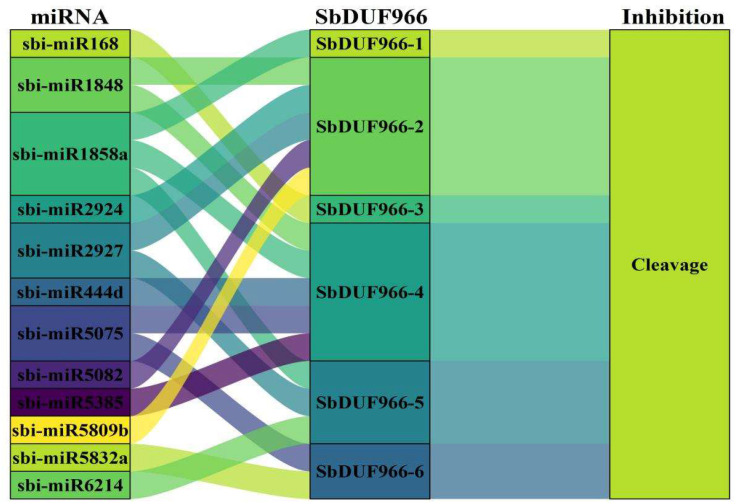
Sankey plot of miRNA targeting in relation to SbDUF966 transcripts.

As depicted in Figure 9, 12 sorghum miRNAs targeting six SbDUF966 genes (SbDUF966-1 to SbDUF966-6) regulate the expression of these genes through cleavage effects. In particular, SbDUF966-2 and SbDUF966-4 were regulated by the targeting of five miRNAs, respectively, with SbDUF966-2 being targeted by sbi-miR1848, sbi-miR2924, sbi-miR2927, sbi-miR5082, and sbi-miR5809b, and SbDUF966-4 being targeted by targeted by sbi-miR1848, sbi-miR1858a, sbi-miR444d, sbi-miR5075, and sbi-miR5385. This highlights that multiple miRNAs interact with the SbDUF966 gene, forming a regulatory network that modulates its expression at the post-transcriptional level.

### 3.8. Expression Profile of SbDUF966 in Different Tissues

**Figure 10:**
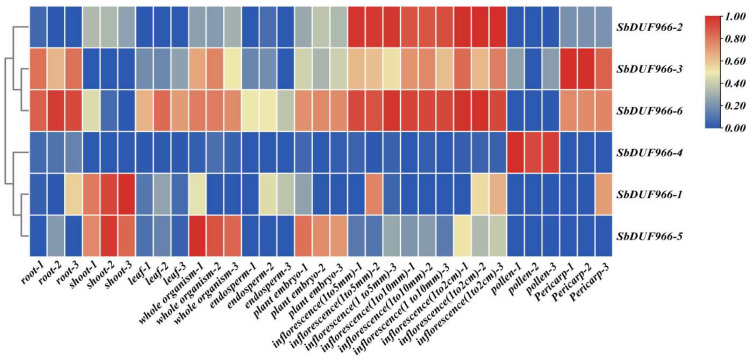
Expression profiles of six SbDUF966 in 11 tissues based on the ENA database, said 11 tissues include roots, stems, leaf, seedlings, pollen, endosperm, embryo, inflorescences (1–5 mm, 1–10 mm, and 1–2 cm), and pericarp.

Gene expression is a key bridge between the transmission of genetic information and the realization of function, and genome-wide gene expression analysis provides insights into expression pathways and regulatory mechanisms. To investigate the spatial and temporal expression patterns of the SbDUF966 gene, we downloaded RNA-seq data from the ENA database (ERP024508 and PRJNA684417). We explored the expression levels of the SbDUF966 gene in different tissues, including roots, stems, leaves, seedlings, pollen, endosperm, embryo, inflorescence (1–5 mm, 1–10 mm, and 1–2 cm), and pericarp. As shown in Figure 9, most SbDUF966 genes had expression in at least one tissue. Among them, SbDUF966-2, SbDUF966-3, and SbDUF966-6 were generally expressed more in reproductive growth than in nutritional growth. In addition, the SbDUF966-4 gene showed high expression in pollen, whereas most of the SbDUF966 genes had low or no expression in pollen (Figure 10).

### 3.9. Analysis of SbDUF966 Gene Expression Under Abiotic Stresses

**Figure 11:**
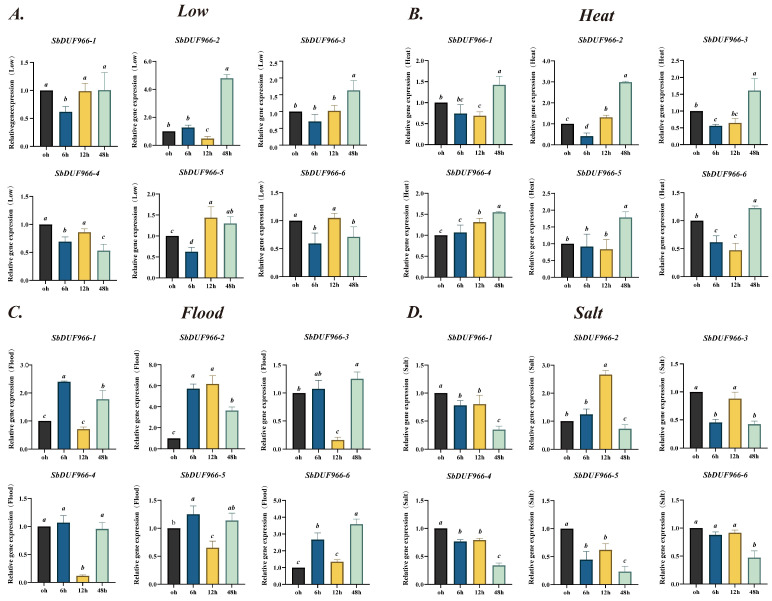
Relative expression profiles of SbDUF966 gene in sorghum under different abiotic stresses. Low temperature (**A**); high temperature (**B**); flooding (**C**); salt (**D**). Each bar represents the mean of 3 replicates, and error bars represent standard deviation (SD). Lowercase letters indicate significant differences at the 0.05 level.

In order to further investigate the expression pattern of the SbDUF966 gene under different abiotic stresses, this study analyzed the expression pattern under four different abiotic stress conditions, including low-temperature stress (Low), heat stress (Heat), flooding stress (Flood), and salt stress (Salt) using RT-qPCR (Figure 11). The samples at 0 h were considered the control group for this experiment; the findings indicated that SbDUF966-5 exhibited a significant upregulation trend under low-temperature stress conditions, especially reaching the highest expression level at 12 h, while SbDUF966-2 was most significantly upregulated at 48 h, suggesting that these genes may play an important role in the mid-to-late stage of the low-temperature response. Under heat stress, the expression of SbDUF966-1, SbDUF966-2, SbDUF966-3, SbDUF966-5, and SbDUF966-6 decreased first, then increased, and reached the peak at 24 h, suggesting that there was a time dependence in the regulation of different genes by heat stress. Flooding stress caused a rapid upregulation of SbDUF966-1 and SbDUF966-2 at 12 h, followed by a gradual decline, reflecting their early response characteristics to drought stress. SbDUF966-2 showed a trend of early upregulation and late suppression under salt stress, suggesting that it may play an important role in the early response to salt stress; at the same time, the expression of SbDUF966-1, 4, 5 and 6 was significantly suppressed under salt stress, especially down-regulated at 48 h. The results showed that SbDUF966-1 and SbDUF966-2 were significantly upregulated and downregulated in the early response to drought at 12 h, followed by a gradual decrease. The above results indicated that the SbDUF966 gene family might be involved in different stages of response mechanisms under salt stress, and some of the genes were repressed at the late stage of stress. Taken together, the SbDUF966 gene family showed significant time dependence and stress specificity under different abiotic stresses, among them, SbDUF966-2 showed a strong response to a wide range of stresses, suggesting that it may play a key role in the abiotic stress adaptation process of plants.

## 4. Discussion

Sorghum is native to Africa and ranks as the fifth most significant cereal globally with strong resistance to drought, flooding, salinity, infertility, and heat. As a C_4_ crop, Sorghum possesses high photosynthetic capacity and biological yield and has important economic and ecological values in agricultural production [22,26].

Gene families are composed of multiple genes produced by gene duplication from homologous genes [27]. These genes have significant structural and functional similarities and perform similar biological functions. The DUF966 gene family is a gene family that has been discovered in higher plants in the last few years. Currently, some members of this gene family have been found to play key roles in plant growth and development and stress tolerance [21]. However, studies on the overall role of the DUF966 family in plant development and stress response are still limited and need to be further explored in depth. In this study, we systematically characterized the DUF966 gene family (SbDUF966) in sorghum by bioinformatics methods, resulting in the identification of six *SbDUF966* genes from the sorghum reference genome (BTx623). Compared to other species, seven were in O. sativa, five were in A. thaliana, eight were in cucumber, and 28 were in T. aestivum. This indicates that gene duplication or loss during gene evolution may lead to differences in the number of gene family members [28]. Based on phylogenetic tree analysis of sorghum with A. thaliana, and of O. sativa and T. aestivum, we categorized the SbDUF966 gene family into two groups: Group I contains SbDUF966-1 and SbDUF966-6, and group II contains SbDUF966-2, SbDUF966-3, SbDUF966-4, and SbDUF966-5. All the subcellular localizations of these proteins were predicted to be located in the nucleus or chloroplasts, which is consistent with the subcellular localization of wheat TaDUF966 family members [19], but different from that of the cucumber CsDUF966 family [17], suggesting that there are variations in the subcellular localization of the DUF966 family proteins among different species. In addition, based on the analysis of gene organization and conserved motifs in the SbDUF966 gene family members identified, the results showed that the SbDUF966 gene was highly conserved in exon and intron structure (Figure 3). However, the number of exons and the length of the introns varied among the different members, which might be the source of its functional differentiation. All six members in SbDUF966 were identified as sharing the common DUF966 structural domain and conserved motifs based on the biosignature informatics system, and this structural similarity is consistent with the results previously reported to be observed in the CsDUF966 and OsDUF966 families [19,29]. Further, this suggests a potential role for these genes in response to abiotic and biotic stresses.

Analysis based on protein 3D structural modeling showed that six members of the SbDUF966 family (SbDUF966-1 to SbDUF966-6) have similar tertiary structures, consisting mainly of β-folded sheets and α-helices and containing irregularly coiled regions as connecting fragments. However, there are minor 3D structural differences in the C terminus of these proteins. This is similar to a previous study on the cucumber CsDUF966 gene family members [17].

Homologous genes (HGs) are genes that have similar gene structure and biological functions in different species or different genes in the same species due to a common evolutionary origin. Thus, gene duplication plays a crucial role in determining gene function. In this study, one pair of tandemly duplicated paralogous homologous genes was found in six SbDUF966 genes of sorghum. Also, to investigate the gene homozygosity between sorghum and other plants, we analyzed the DUF966 genes of monocotyledonous plants (*O. sativa*, *Z*. *mays*, and *H*. *vulgare*) as well as dicotyledonous plants (*G*. *max* and *A*. *thaliana*). The results showed that SbDUF966 had 4, 11, 7, and 2 pairs of homologous genes with OsDUF966, ZmDUF966, HvDUF966 and GmDUF966, respectively. This shows that the number of DUF966 homologous genes is considerably greater in monocots compared to dicots. In addition, no DUF966 homologous genes were found in *A*. *thaliana*. This implies that the DUF966 gene family may play a more crucial evolutionary role and have greater biological significance in monocots.

Cis-acting elements in the promoter region of genes are involved in the transcriptional regulation of genes by binding to transcription factors, regulating changes in plant expression during a variety of physiological processes such as growth and development, and response to adversity. Studies have shown that members of the DUF966 family play important roles in plant growth, development, and response to adversity [4,12,20]. We analyzed the cis-acting elements of the SbDUF966 promoter region at 2000 bp upstream of sorghum. We found that the SbDUF966 family genes contain 30 cis-acting elements, which are mainly involved in four categories of functions, including “light response”, “biotic/abiotic stress”, “growth and development”, and “plant hormone response” (Figure 8). It was found that the SbDUF966 family genes contained 30 cis-acting elements, which were mainly involved in four categories of functions, including “light response”, “biotic/abiotic stress”, “growth and development”, and “phytohormone response”. This suggests that the SbDUF966 gene has an important role in the growth and development of plants. The same result was also verified in pepper and T. aestivum DUF966 genes, where cis-acting elements related to growth and development accounted for the most significant proportion [30]. In addition, methyl jasmonate (MeJA) and abscisic acid (ABA) are important phytohormones that are widely involved in a variety of signaling pathways and regulate physiological and molecular processes in plants. These hormones play a central role in defense against biotic stresses as well as mitigation of abiotic stresses such as drought, salt damage, vernalization, and heavy metal exposure [31,32]. In this study, we found that cis-acting elements related to methyl jasmonate (MeJA) and abscisic acid (ABA) were abundantly present in the promoter region of SbDUF966 family genes. This result is in agreement with the results of previous studies on cis-acting elements in the promoter of the wheat TaDUF966 gene [19].

miRNAs (microRNAs) are non-coding small RNAs that regulate plant growth, development, and stress responses by targeting mRNAs, leading to cleavage or translation repression [33]. We explored the regulatory interactions at the post-transcriptional level between miRNAs and SbDUF966 transcripts. As illustrated in Figure 8, in this study, by predictive analysis, we identified 12 sorghum miRNAs targeting six SbDUF966 genes and regulating their expression through cleavage effects. For example, sbi-miR1848, sbi-miR2924, sbi-miR2927, sbi-miR5082, and sbi-miR5809b target SbDUF966-2, whereas sbi-miR1848, sbi-miR1858a, sbi-miR444d, sbi-miR5075, and sbi miR5385 target SbDUF966-4. miRNAs such as sbi-miR1848 play key roles in plant growth, development, and response to adversity. In the current study, in rice, sbi-miR1848 regulates sterol and BR biosynthesis in plants by targeting OsCYP51G3 and responds to adversity signaling by regulating its expression under salt stress [34].

Many studies have shown that DUF family members are associated with plant stress tolerance [35,36]. For example, in tobacco, NtDUF668-03 was transcriptionally upregulated after 6 h of salt treatment. However, its expression level dropped sharply after 12 h of salt exposure, implying the presence of a feedback regulation system for salt adaptation and stress responses [37]; in A. thaliana, the AtDUF506 family, which is widely present in photosynthetic organisms, shows potential roles in coping with environmental stress and nutrient shortage [36]. In rice, OsDUF1664.3 enhances ROS scavenging enzyme activity and improves drought resistance in transgenic *E. coli* [38]. In this study, to further investigate the expression pattern of SbDUF966 genes under abiotic stress, we performed qRT-PCR analysis of six SbDUF966 genes, and the results showed (Figure 11) that under low-temperature stress conditions, the expression of SbDUF966-5 peaked at 12 h, whereas SbDUF966-2 was significantly upregulated at 48 h, indicating that these genes may play a role in the middle and late stages of low-temperature response. Under heat stress, SbDUF966-1, 2, 3, 5, and 6 showed a tendency of decreasing and then increasing and reached the peak at 24 h, indicating the time-dependence of the regulation of heat stress. Flooding stress induced a rapid upregulation of SbDUF966-1 and 2 at 12 h, followed by a gradual decrease, reflecting its early response characteristics to stress. Under salt stress, SbDUF966-2 showed early upregulation and late inhibition, suggesting its key role in the early response to salt stress. At the same time, SbDUF966-1, 4, 5, and 6 expression was significantly inhibited. A significant decline was mainly observed at 48 h. These results suggest that the SbDUF966 gene family exhibits distinct time-dependent and stress-specific responses to various abiotic stresses, with SbDUF966-2 demonstrating a stronger response to multiple stresses, indicating its potential role in plant adaptation to abiotic stress. To further validate the role of the SbDUF966 gene family in stress responses, future studies should focus on investigating its relationship with stress response mechanisms, phenotypic variations, and physiological changes.

Based on the above results, the identification of the DUF966 gene family in the sorghum genome and its response to multiple abiotic stresses suggest that this gene family plays an important role in plant stress response. These findings provide valuable insights into the potential functions of the SbDUF966 genes, thereby promoting the breeding of new stress-tolerant varieties.

## 5. Conclusions

Six SbDUF966 genes in sorghum were identified by bioinformatics analysis and categorized into two subgroups (Group I and Group II). Subcellular localization predictions indicated that these genes are predominantly located in the nucleus. Analysis of gene structure, chromosomal distribution, and evolution showed that the SbDUF966 gene family is highly conserved throughout sorghum evolution. Promoter analysis revealed numerous cis-acting elements related to growth, development, and hormone regulation. Expression patterns and miRNA analyses suggested the regulatory involvement of the SbDUF966 gene family in sorghum growth and development. RT-qPCR further confirmed that the expression of SbDUF966 genes under abiotic stresses is time-dependent and stress-specific, suggesting that they may play an important role in sorghum adaptation to environmental stresses.

## Figures and Tables

**Table 1:** Physicochemical characteristics and predicted subcellular localization of sorghum SbDUF966.

Name	Gene ID	Number of Amino Acid (aa)	Molecular Weight (KD)	Theoretical pI	Instability Index	Aliphatic Index	Grand Average of Hydropathicity	Subcellular Localization
SbDUF966-1	XM_002464013.2	Chr01	556	62,625.16	8.73	71.71	63.65	−0.851
SbDUF966-2	XM_002465667.2	Chr01	374	40,953.67	7.16	61.77	60.32	−0.805
SbDUF966-3	XM_021453260.1	Chr02	587	63,305.47	8.6	65.47	69.45	−0.53
SbDUF966-4	XM_002456393.2	Chr03	441	48,779.1	9.33	66.88	48.28	−1.138
SbDUF966-5	XM_002458689.2	Chr03	410	44,266.75	9.44	53.53	70.8	−0.548
SbDUF966-6	XM_021465023.1	Chr07	615	66,240.58	7.61	69.24	61.72	−0.692

## Data Availability

The RNA-seq data used in this study are available in the NCBI-SRA database (ERP024508 and PRJNA684417). Additional data supporting the findings can be found in the Supplementary Materials.

## Supplementary Materials

The following supporting information can be downloaded at: https://www.mdpi.com/article/10.3390/genes16020206/s1, Figure S1: Attachment 1 Amplification Curve; Table S1: RT-qPCR primer information; Table S2: Expression profiles of different tissues; Table S3: RT-qPCR data for different abiotic stresses; Table S4: miRNA targeting SbDUF966.
